# Supplementary material for: Cryo-EM structures of the human P2X1 receptor reveal subtype-specific architecture and antagonism by supramolecular ligand-binding
Source: Nat Commun. 2024 Oct 1;15:8490. doi: 10.1038/s41467-024-52636-4 (PMC11448502; doi:10.1038/s41467-024-52636-4)
Supplement: Supplementary file 1 — Supplementary Information [file 41467_2024_52636_MOESM1_ESM.pdf]

Cryo-EM structures of the human P2X1 receptor reveal subtype-specific architecture and antagonism by supramolecular ligand-binding

Authors: Adam C. Oken[a,†], Nicolas E. Lisi[a,†], Ismayn A. Ditter[a,†], Haoyuan Shi[a,†], Nadia A. Nechiporuk[a], and Steven E. Mansoor[a,b,*]

Affiliations:

a. Department of Chemical Physiology & Biochemistry, Oregon Health & Science University, Portland, Oregon 97239, USA.

b. Division of Cardiovascular Medicine, Knight Cardiovascular Institute, Oregon Health & Science University, Portland, Oregon 97239, USA.

† These authors contributed equally

* To whom correspondence should be addressed: mansoors@ohsu.edu

This PDF file includes:

Supplementary Figures 1 to 12
Supplementary Table 1

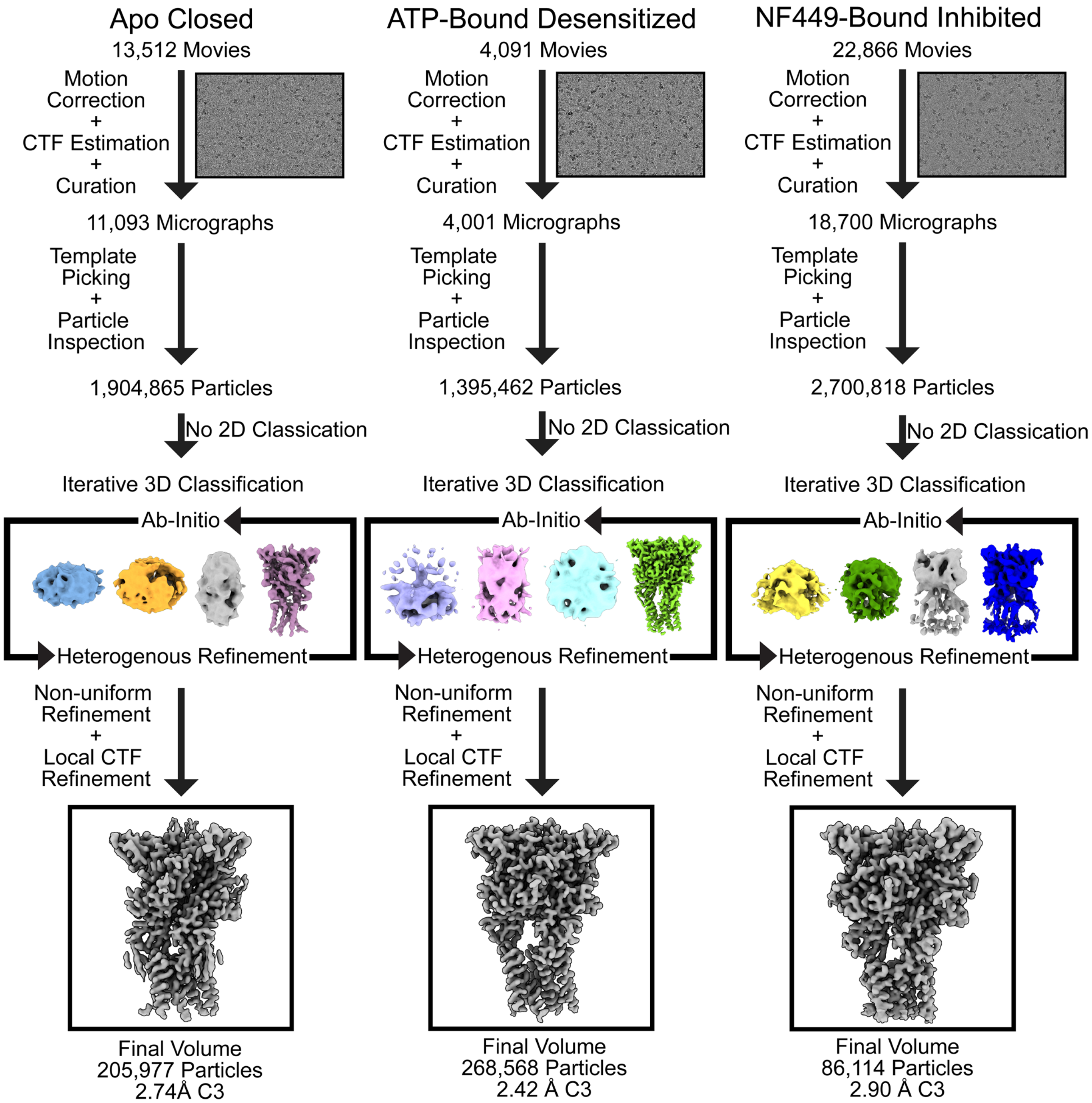

Supplementary Fig. 1: Cryo-EM processing pipeline for hP2X1 reconstructions. Cryo-EM image processing strategies for the apo closed, ATP-bound desensitized, and NF449-bound inhibited states using cryoSPARC[1]. After data acquisition, movies were motion corrected, CTF parameters estimated, curated, and template picked. Particles were inspected, extracted, and sent directly to iterative 3D classification (skipping 2D classification) using ab-initio jobs to generate initial reconstructions that composed the inputs of heterogenous classifications. After final particle stacks were obtained, further CTF corrections and non-uniform refinements were performed at the physical pixel size to generate the final reconstructions.

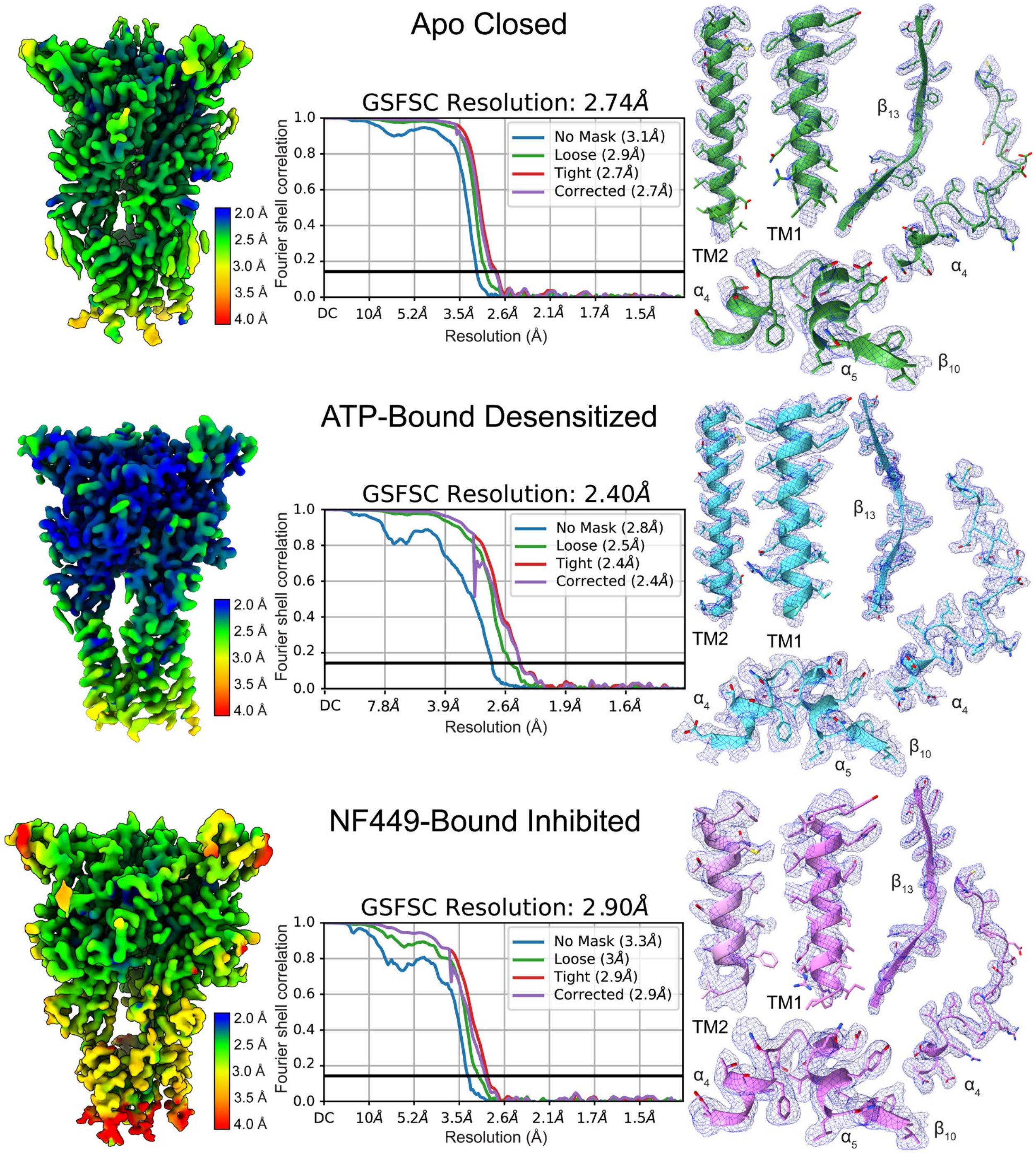

Supplementary Fig. 2: Local resolution, Fourier shell correlation (FSC) plots, and map-to-model fit for the cryo-EM maps of hP2X1 receptor in the apo closed, ATP-bound desensitized, and NF449-bound inhibited states. The resolutions stated are at an FSC = 0.143. All local resolution plots range from 2.0 Å (blue) to 4.0 Å (red). Different views of the apo closed (green ribbon and blue mesh), ATP-bound desensitized (blue ribbon and blue mesh), and NF449-bound inhibited (pink ribbon and blue mesh) states highlight good map-to-model fits.

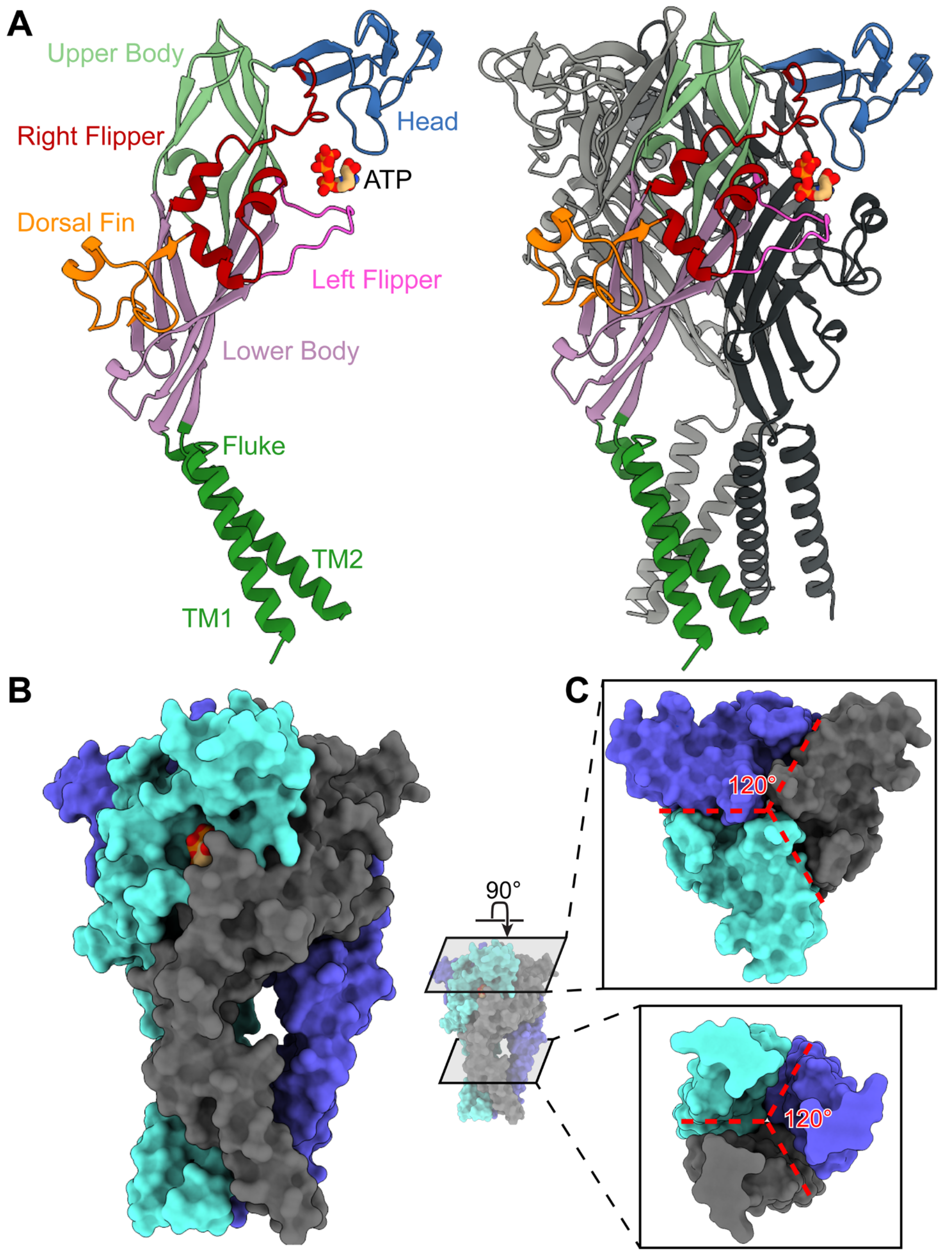

Supplementary Fig. 3: Naming of the purinergic receptor domains. (A) Ribbon representation of one protomer (*left*) and a full trimer (*right*) of hP2X1 in the ATP-bound desensitized state, colored by domain. The second and third protomers of the trimeric receptor are colored in light gray and dark gray, respectively. **(B)** Space filling model of hP2X1 in the ATP-bound desensitized state shown parallel to the membrane. **(C)** Space filling model of hP2X1 in the ATP-bound desensitized state shown perpendicular to the membrane as a top-down view from the extracellular domain ***(top)*** and from the center of the TMD ***(bottom).*** These images highlight how the three protomers intertwine through domain swapping such that each protomer undergoes a relative ~120° rotation from the extracellular domain to the TMD.

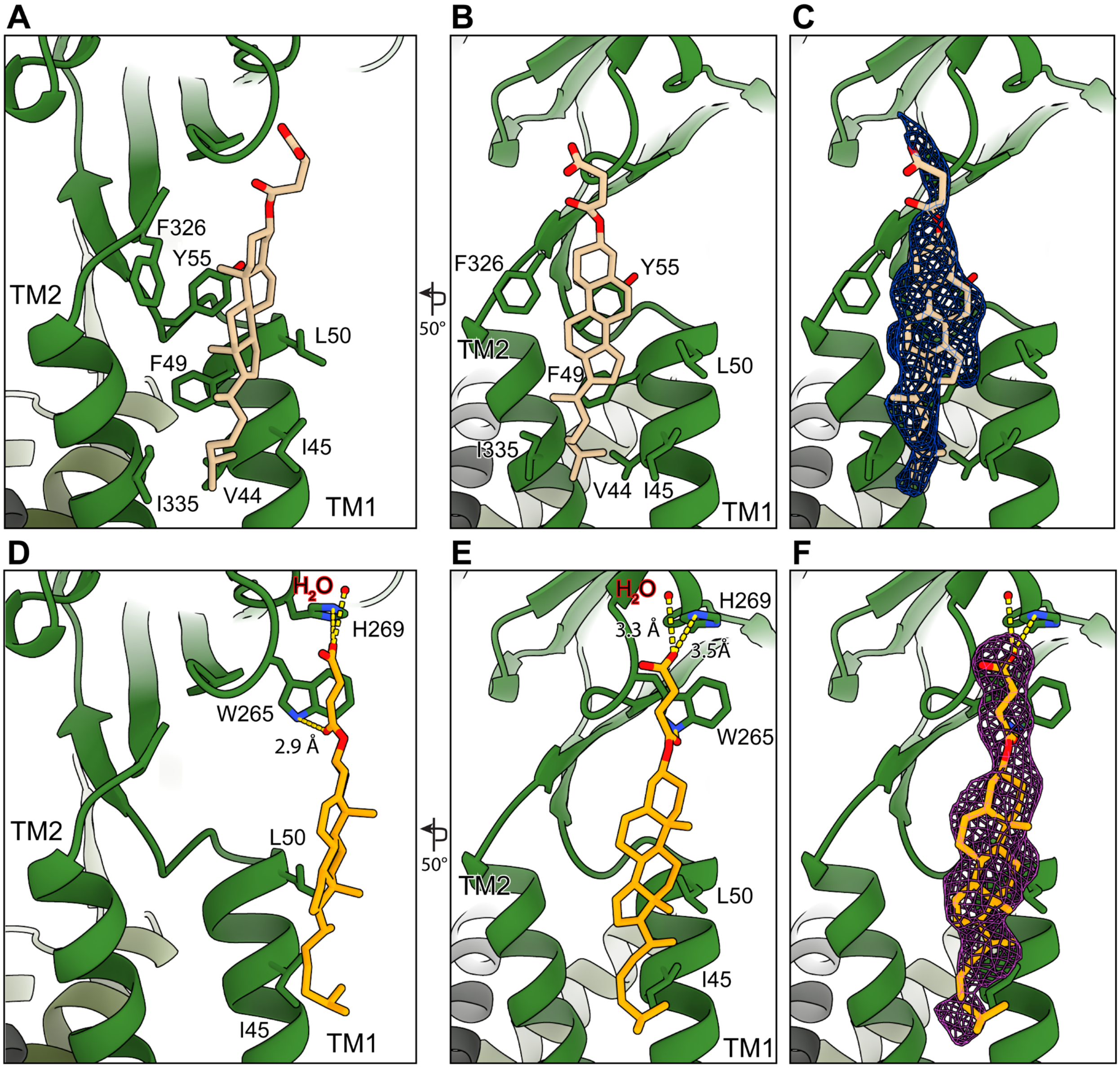

Supplementary Fig. 4: Cholesterol hemi-succinate (CHS) binds to hP2X1 at the interface between TM1 and TM2. (A-C) Ribbon representation of the inner CHS (tan) molecule which binds to hP2X1 at the interface between TM1 and TM2 on the extracellular leaflet of the membrane. For clarity, the outer CHS molecule is hidden. **(A)** Magnified view of Fig. 1B highlighting the hydrophobic interactions between the receptor and the inner CHS molecule. **(B)** A 50° rotated view of panel A highlighting the position of the inner CHS between TM1 and TM2. **(C)** Same view as panel B with the cryo-EM density for the inner CHS molecule shown in dark blue mesh. **(D-F)** Ribbon representation of the outer CHS (orange) molecule which binds to hP2X1 at the interface between TM1 and TM2 on the extracellular leaflet of the membrane. This molecule predominantly interacts with TM1 and the inner CHS molecule. For clarity, the inner

CHS molecule is hidden. **(D)** Magnified view of Fig. 1B highlighting the interactions between the receptor and the outer CHS molecule (orange). This view highlights the hydrogen bond between the outer CHS molecule and the sidechain nitrogen of W265. **(E)** A 50° rotated view of panel D, highlighting the interactions between the succinate group on the outer CHS molecule and the sidechain of H269, as well as a water molecule. **(F)** Same view of panel E with the cryo-EM density for the outer CHS molecule shown in dark purple mesh.

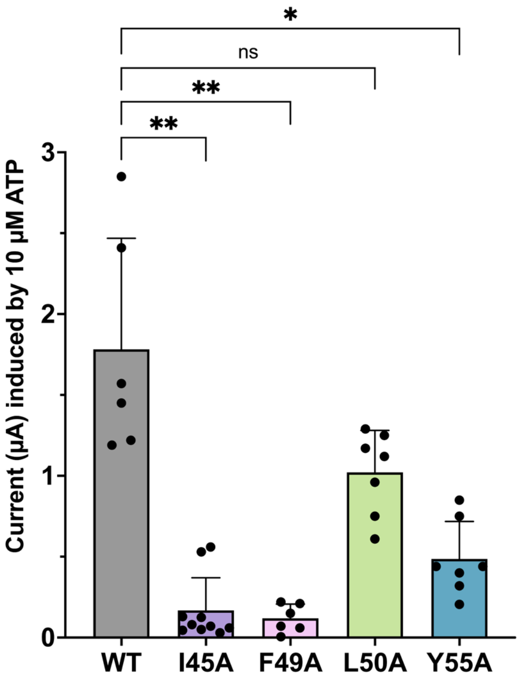

Supplementary Fig. 5: Mutation of residues that coordinate CHS molecules affect the magnitude of ATP-induced currents. Receptors expressed in oocytes were potentiated and activated by 10 µM ATP to determine maximum current for each receptor construct. Mutations I45A, F49A, and Y55A significantly decreased the magnitude of ATP-induced currents, suggesting these residues are important to the function of hP2X1. In contrast, the mutation L50A did not significantly impact the magnitude of ATP-induced currents, suggesting this residue is not critical for receptor function. Statistical analysis of current amplitudes was performed using a Dunnett T3 ANOVA test. When comparing maximal currents to wild-type hP2X1 (gray), the effects of mutations I45A (purple) and F49A (pink) were highly significantly different (**, $p < 0.01$), Y55A (blue) was significantly different (*, $p < 0.05$), and L50A (green) was not significantly different (ns, $p > 0.05$). Data represents mean and standard deviations (n = 6-10).

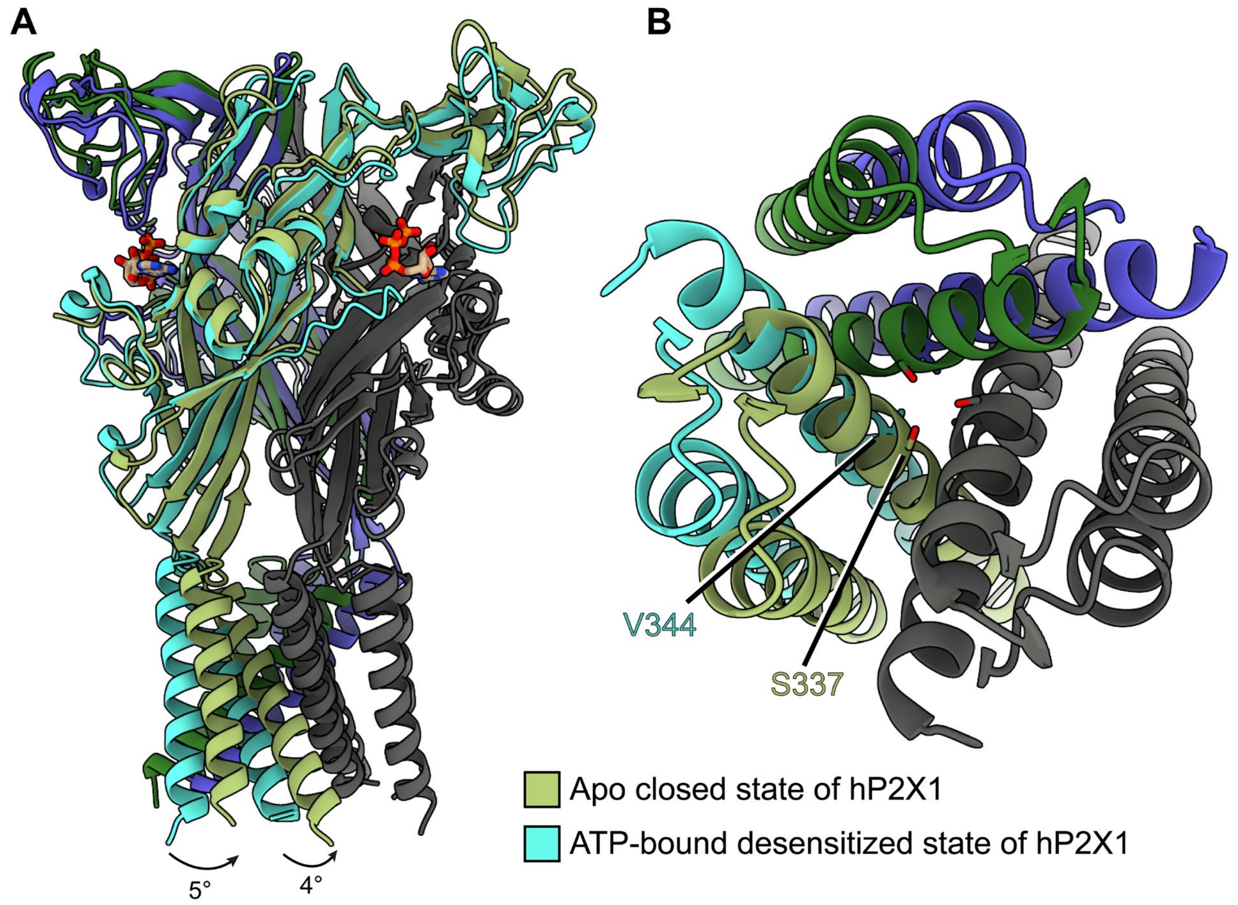

Supplementary Fig. 6: Comparison between the apo closed and ATP-bound desensitized states of hP2X1. (A) Overlayed structures of hP2X1 in the apo closed (shades of green and gray) and ATP-bound desensitized (shades of blue and gray) states, highlighting the differences between the two conformations, especially in the TMD. **(B)** Top-down view of the transmembrane domain showing the differences in helical pitch between apo closed (shades of green) and ATP-bound desensitized (shades of blue) state structures of hP2X1. The constriction gate in the apo closed state of hP2X1, formed by S337 from each protomer, is 7 Å deep into the plasma membrane. In contrast, the constriction gate in the ATP-bound desensitized state structure of hP2X1, formed by V344 from each protomer, is 16 Å deep into the plasma membrane.

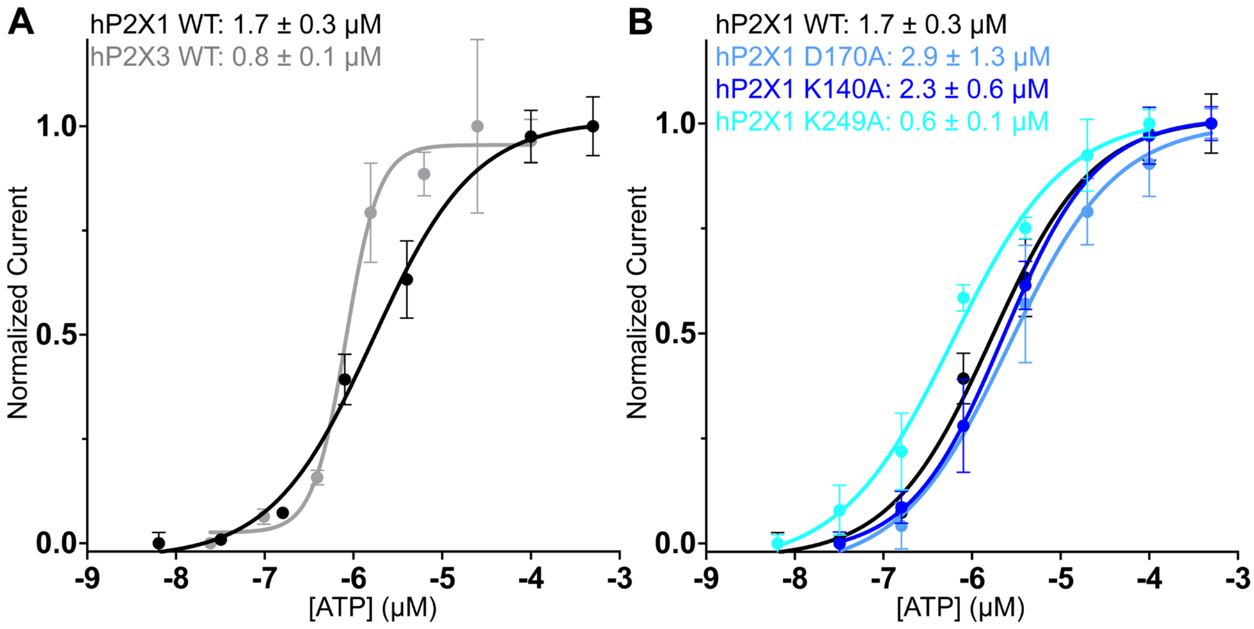

Supplementary Fig. 7: Effects of mutations on the apparent affinity (EC_{50}) of ATP to hP2X1. (A) Dose-response curves (EC_{50}) for the activation of wild-type hP2X1 (black) and wild-type hP2X3 (gray) by ATP. Activation of hP2X3 by ATP appears to show greater cooperativity than activation of hP2X1 by ATP, with hill coefficients of 2.5 ± 0.6 compared to 0.9 ± 0.2, respectively. Hill coefficient values represent the mean and standard deviation from three discrete EC_{50} traces. (B) Dose-response curves (EC_{50}) for the activation of wild-type (black) and mutant (shades of blue) hP2X1 by ATP highlighting that mutation of residues specific to hP2X1 do not significantly alter the apparent affinity of ATP. The mutants D170A, which removes the residue that coordinates a Mg^{2+} ion in the orthosteric pocket, K140A, which removes a subtype-specific interaction with the ribose of ATP, and K249A, which alters the electrostatics surrounding the orthosteric pocket, each do not appear to significantly change the apparent affinity of ATP for hP2X1. For all traces, the Y-axis describes currents normalized to the largest current evoked by the maximum concentration of ATP applied to each oocyte. The reported EC_{50} and error bars represent the mean and standard deviation across triplicate experiments, respectively.

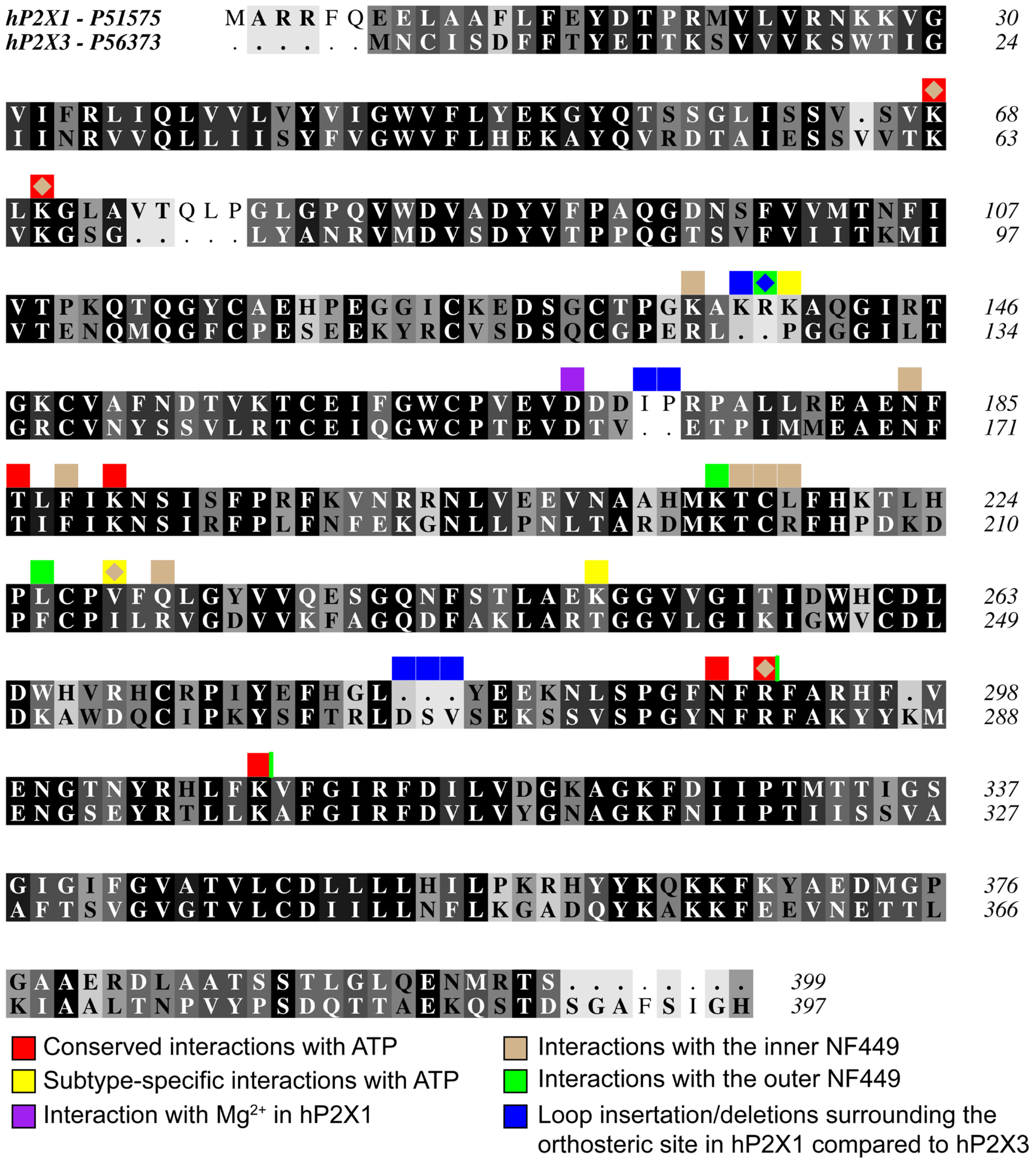

Supplementary Fig. 8: Sequence alignment of hP2X1 and hP2X3. Protein sequence alignment of hP2X1 and hP2X3 shown in grayscale by level of sequence conservation, as calculated by Alscript (black squares denote conservation and white squares denote no conservation)[2]. Important residues are indicated by squares, diamonds, and right bars above the sequence alignment and colored according to their functional role. Red coloring represents the seven conserved residues across all P2X receptors that interact with ATP. Yellow coloring

represents subtype-specific residues that interact with ATP. Purple coloring represents the residue in hP2X1 that interacts with the Mg^{2+} ion. Tan coloring represents residues that interact with the inner molecule of NF449. Green coloring represents residues that interact with the outer molecule of NF449. Blue coloring represents insertion or deletions within hP2X1 surrounding the orthosteric pocket compared to hP2X3. Sequences were obtained from UniProt with accession codes P51575 (hP2X1) and P56373 (hP2X3) and the alignment was performed in Clustal Omega[3]. The figure was generated with Aline[4].

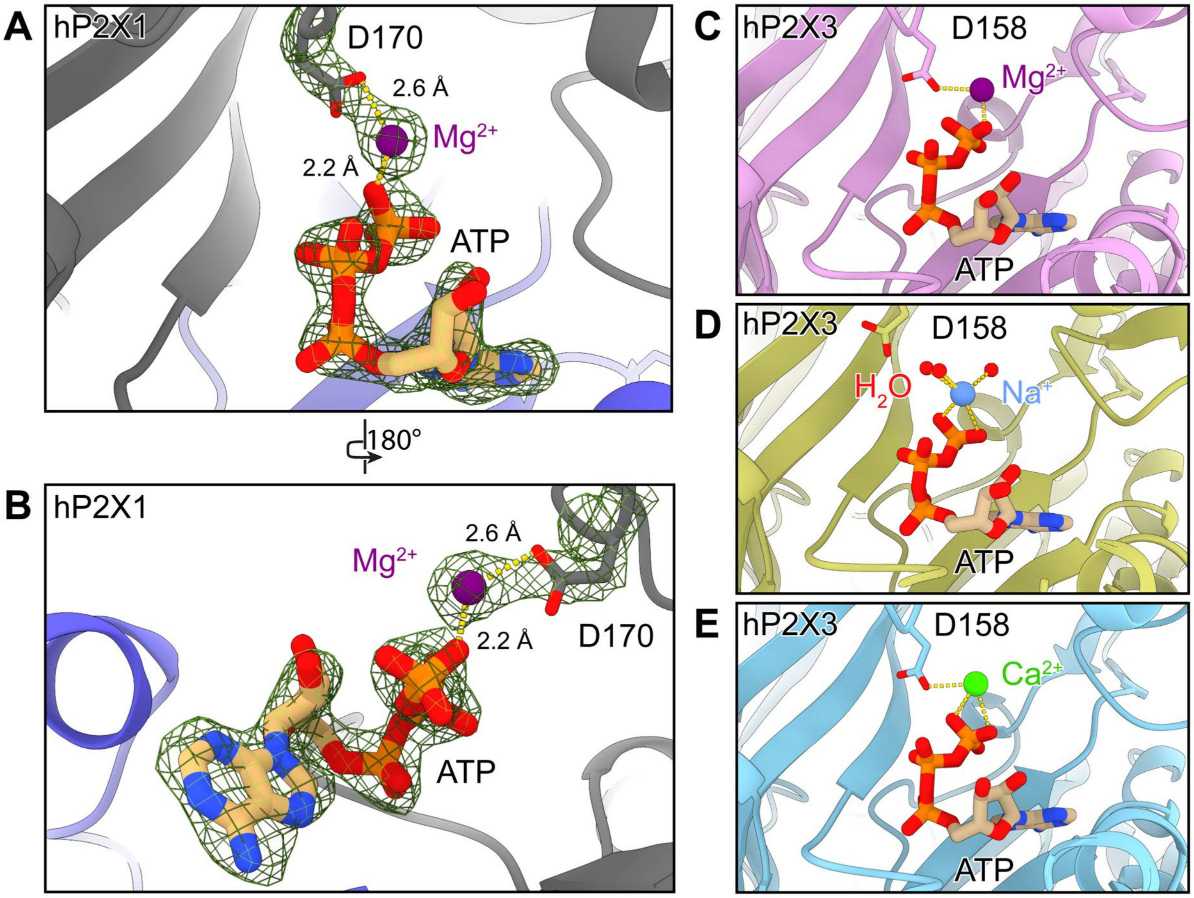

Supplementary Fig. 9: Cryo-EM density for ATP and the Mg^{2+} ion bound within the orthosteric pocket in the ATP-bound desensitized state structure of hP2X1. (A and B) Magnified views of the orthosteric pocket in hP2X1 highlighting the interactions between the γ-phosphate of ATP, the Mg^{2+} ion, and residue D170. Additionally, the cryo-EM density (green mesh) is shown for ATP, the Mg^{2+} ion, and residue D170. **(C-E)** Same view as panel A highlighting the coordination of ATP in hP2X3 structures to a Mg^{2+} ion (C), a Na^{+} ion (D), and a Ca^{2+} ion (E) (PDB codes: 6AH5, 5SVL 6AH4, respectively)[5,6]. The residue that coordinates Mg^{2+} in hP2X1 is D170 which corresponds to D158 in hP2X3 (Supplementary Fig. 8). These panels highlight that the sidechain of D170 in hP2X1 is positioned differently than the sidechain of D158 in hP2X3, likely the result of the two-residue insertion found within the loop containing D170 in hP2X1.

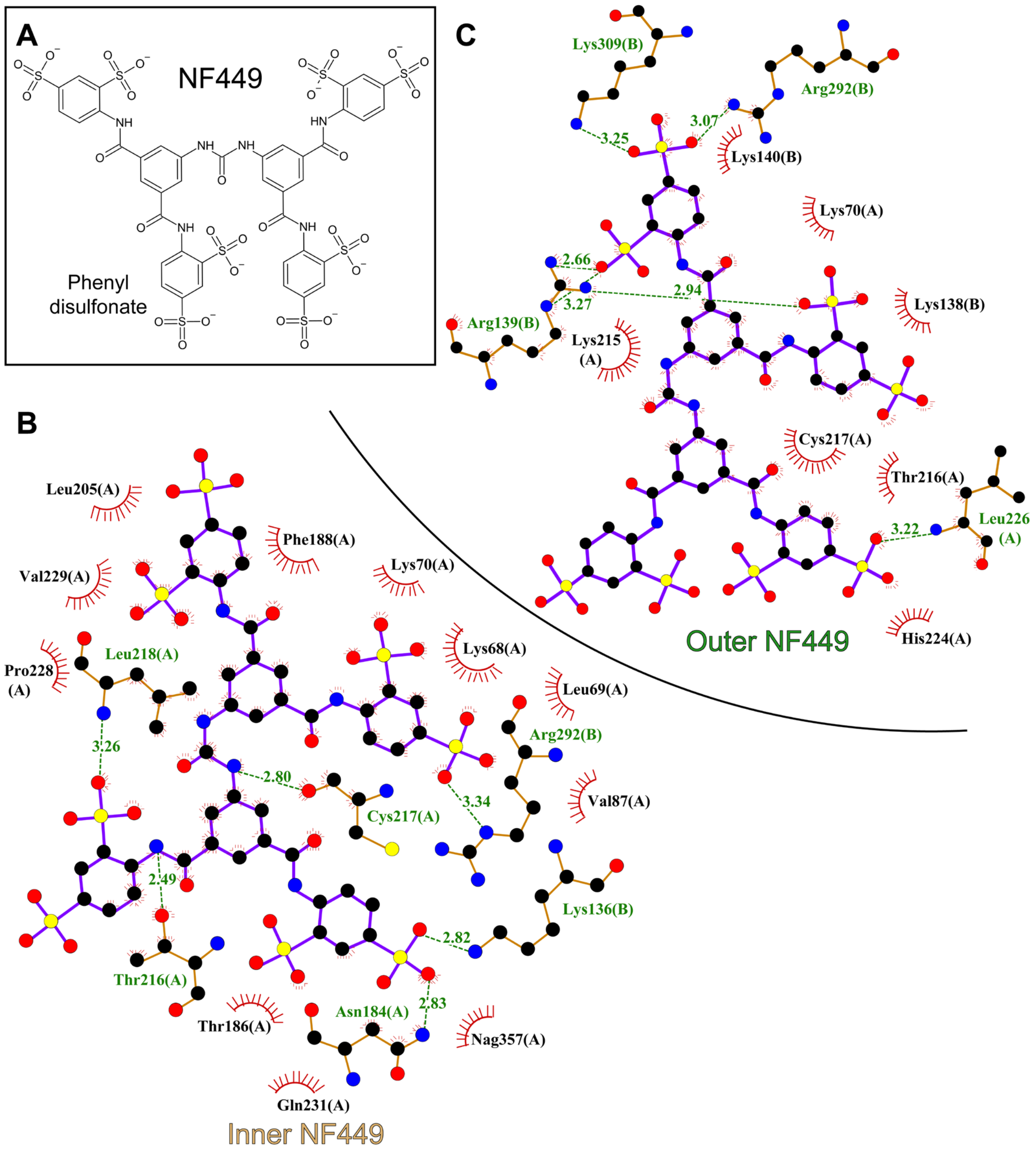

Supplementary Fig. 10: Chemical structure of NF449 and LigPlot for both molecules of NF449 bound to hP2X1. (A) 2D-chemical structure of NF449. **(B)** LigPlot diagram for the inner NF449 molecule highlighting a plethora of interactions, including interactions with K136 and residues within the orthosteric ATP-binding site such as K68, K70, and R292. **(C)** LigPlot diagram for the outer NF449 molecule highlighting interactions with R139 and R292[7].

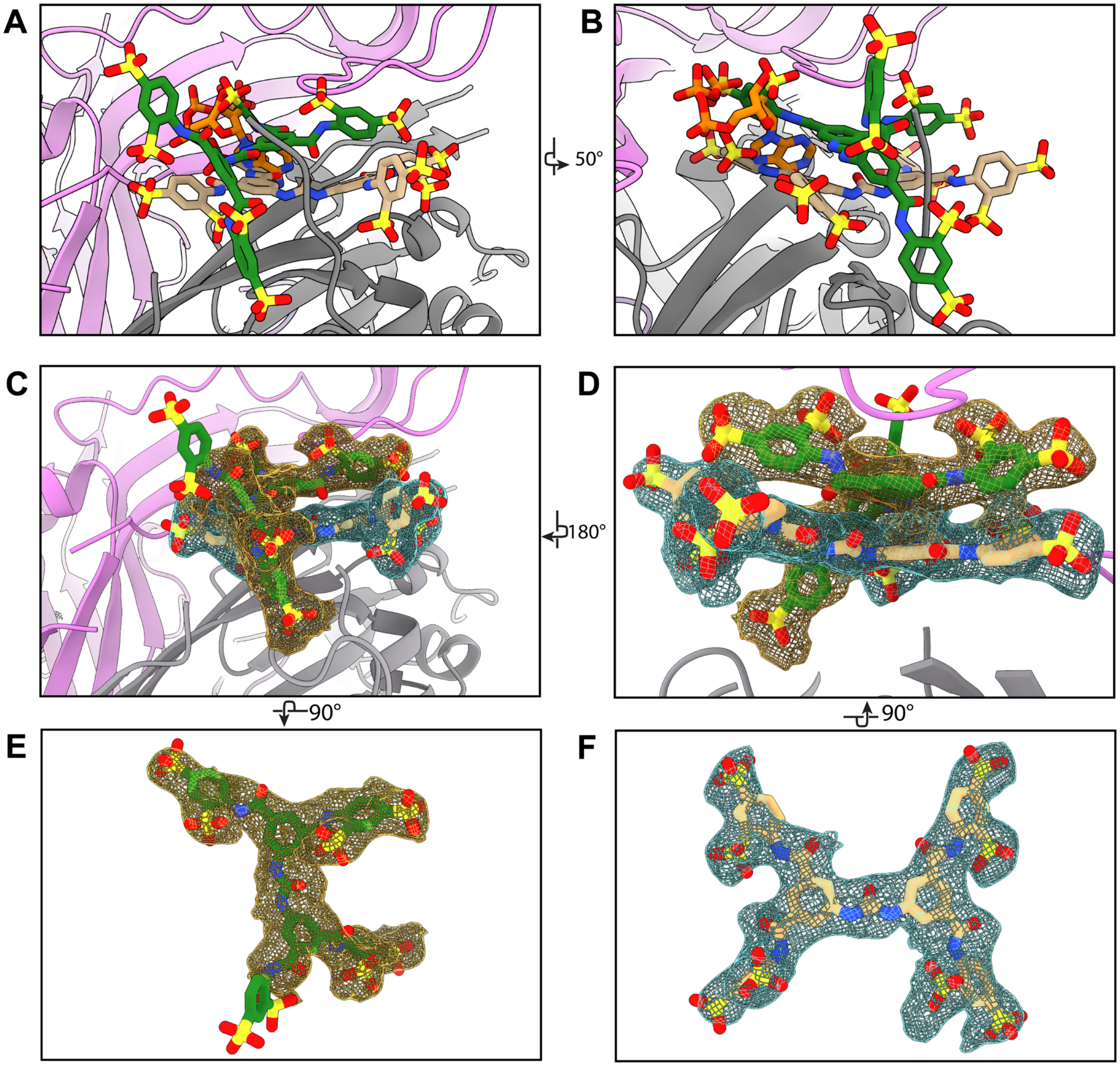

Supplementary Fig. 11: Cryo-EM density of the inner and outer molecules of NF449 when bound to hP2X1. (A) Same view of the NF449 binding pocket as shown in Fig. 5B but with the ATP molecule (dark orange) from the ATP-bound desensitized state structure overlayed, highlighting how parts of both NF449 molecules (inner colored in tan and outer colored in green) occupy the orthosteric binding pocket. **(B)** A 50° rotated view from panel A showing a second perspective of moieties from both NF449 molecules occupying the same location as ATP within the orthosteric pocket. **(C)** Same view as panel A with the inner (tan) and outer (green) molecules of NF449 shown in their respective cryo-EM densities in aqua and brown mesh, respectively. **(D)** A 180° rotated view of panel C highlighting the stacked nature of the two NF449 molecules, with distinct density for both molecules. **(E)** A 90° rotated view of panel C

highlighting the cryo-EM density (brown mesh) for the outer (green) molecule of NF449. For the outer ligand, three arms of NF449 have good cryo-EM density fit, while the fourth arm, which is farthest from the receptor and lacks interactions, has poor density fit due to high flexibility. **(F)** A 90° rotated view of panel D highlighting the good cryo-EM density (aqua mesh) for the entire inner (tan) molecule of NF449.

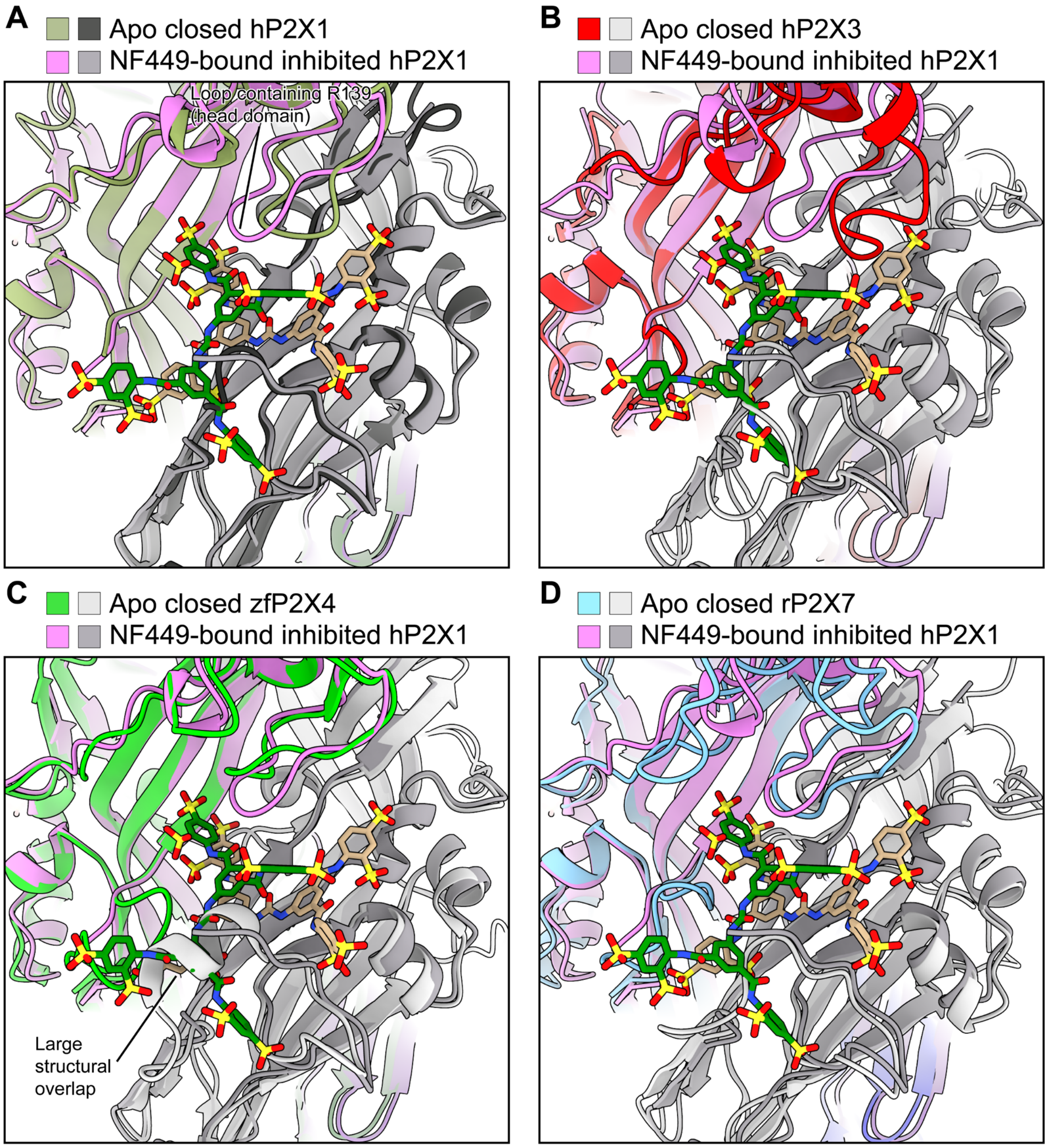

Supplementary Fig. 12: Subtype-specific structural features of the NF449 competitive ligand-binding site. (A) Overlayed structures of hP2X1 in the apo closed state (olive and dark gray) and hP2X1 in the NF449-bound inhibited state (pink and gray) highlighting the rearrangements of extracellular loops between the two states that occur to accommodate NF449 binding. The loop containing R139 in the head domain shifts to interact with the outer NF449 molecule (green) (Supplementary Fig. 3A). **(B)** Overlayed structures of hP2X3 in the apo closed state (red and light gray, PDB code: 5SVJ) and hP2X1 in the NF449-bound inhibited state (pink and gray), highlighting dramatic structural differences between loops in the head domain (Supplementary Fig. 3A)[5]. **(C)** Overlayed structures of zfP2X4 in the apo closed state

(green and light gray, PDB code: 4DW0) and hP2X1 in the NF449-bound inhibited state (pink and gray) highlighting the overlap between an alpha helix in the dorsal fin domain and both molecules of NF449 (Supplementary Fig. 3A)[8]. **(D)** Overlayed structures of rP2X7 in the apo closed state (light blue and light gray, PDB code: 6U9V) and hP2X1 in the NF449-bound inhibited state (pink and gray) highlighting similar pockets with minimal structural differences[9].

Supplementary Table 1: Cryo-EM collection, refinement, and validation statistics.

	APO hP2X1 (EMD-45152) (PDB: 9C2A)	ATP-bound hP2X1 (EMD-45153) (PDB: 9C2B)	NF449-bound hP2X1 (EMD-45154) (PDB: 9C2C)
**Data collection and processing**			
Magnification (kx)	130	130	130
Voltage (kV)	300	300	300
Electron exposure (e–/Å^2)	45	44	45
Movie frames	50	48	50
Defocus range (μm)	-0.8 to -1.4	-0.8 to -1.4	-0.9 to -1.4
Pixel size (Å)	0.6488	0.648 (0.324 super-res)	0.6488
Symmetry imposed	C3	C3	C3
Initial micrographs (no.)	13,512	4,091	22,866
Final micrographs used (no.)	11,093	4,001	18,700
Initial particle images (no.)	1,904,865	1,395,462	2,700,818
Final particle images (no.)	205,977	268,568	86,114
Map resolution (Å)	2.74	2.42	2.90
FSC threshold	(0.143)	(0.143)	(0.143)
Map resolution range (Å)	1.45 - 25.2	1.46 - 29.6	1.78 - 35.5
**Refinement**			
Initial model used (PDB code)	Homology model: 5SVJ	Homology model: 5SVL	Homology model: 5SVJ
Model resolution (Å)	2.73	2.41	2.85
FSC threshold	(0.143)	(0.143)	(0.143)
Map sharpening *B* factor (Å^2)	108.8	76.2	87.8
**Model composition**			
Non-hydrogen atoms	7,833	8208	7,593
Protein Residues	933	990	885
Ligands	21	24	15
Waters	102	141	0
B factors (Å^2)			
Protein	38.3/108/56.0	14.5/109/39.0	30.0/118/57.8
Ligand	49.8/117/68.8	18.3/52.9/29.5	55.6/82.8/57.8
Nucleotide	--	--	--
Water	41.6/74.2/52.1	13.0/87.4/25.6	--
R.m.s. deviations			
Bond lengths (Å)	0.008 (0)	0.007 (0)	0.007 (0)
Bond angles (°)	0.910(0)	0.936 (0)	0.924 (0)
Validation			
MolProbity score	1.45	1.20	1.66
Clash score	5.95	3.77	5.49
Poor rotamers (%)	0.00	0.00	0.00
Ramachandran plot			
Favored (%)	97.38	97.87	94.77
Allowed (%)	2.62	2.13	5.23
Disallowed (%)	0.00	0.00	0.00

References:

1. Punjani, A., Rubinstein, J.L., Fleet, D.J. & Brubaker, M.A. cryoSPARC: algorithms for rapid unsupervised cryo-EM structure determination. *Nat Methods* **14**, 290-296 (2017).
2. Barton, G.J. ALSCRIPT: a tool to format multiple sequence alignments. *Protein Engineering, Design and Selection* **6**, 37-40 (1993).
3. Sievers, F. & Higgins, D.G. Clustal Omega for making accurate alignments of many protein sequences. *Protein Sci* **27**, 135-145 (2018).
4. Bond, C.S. & Schuttelkopf, A.W. ALINE: a WYSIWYG protein-sequence alignment editor for publication-quality alignments. *Acta Crystallogr D Biol Crystallogr* **65**, 510-2 (2009).
5. Mansoor, S.E. et al. X-ray structures define human P2X(3) receptor gating cycle and antagonist action. *Nature* **538**, 66-71 (2016).
6. Li, M. et al. Molecular mechanisms of human P2X3 receptor channel activation and modulation by divalent cation bound ATP. *eLife* **8**, e47060 (2019).
7. Laskowski, R.A. & Swindells, M.B. LigPlot+: Multiple Ligand–Protein Interaction Diagrams for Drug Discovery. *Journal of Chemical Information and Modeling* **51**, 2778-2786 (2011).
8. Hattori, M. & Gouaux, E. Molecular mechanism of ATP binding and ion channel activation in P2X receptors. *Nature* **485**, 207-12 (2012).
9. McCarthy, A.E., Yoshioka, C. & Mansoor, S.E. Full-Length P2X(7) Structures Reveal How Palmitoylation Prevents Channel Desensitization. *Cell* **179**, 659-670 e13 (2019).
